# Impact pathways of adverse childhood experiences on infectious diseases among substance abusers in border regions: structural equation modeling

**DOI:** 10.3389/fpubh.2025.1518607

**Published:** 2025-05-09

**Authors:** Mingmei Zhang, Jianhui He, J. Melvin Young, Jing You, Jing Li

**Affiliations:** ^1^Yunnan Provincial Key Laboratory of Public Health and Biosafety, Kunming Medical University, Kunming, China; ^2^School of Public Health, Kunming Medical University, Kunming, China; ^3^Department of Infectious Diseases and Hepatology, The First Affiliated Hospital of Kunming Medical University, Kunming, China

**Keywords:** injection of drug use, adverse childhood experiences, infectious disease, structural equation models, border

## Abstract

**Background:**

Injection of drug abuse could result in infectious disease, and adverse childhood experiences (ACEs) possibly are associated with infectious disease. However, there is a paucity of literature on a direct or indirect relationship between ACEs, injection of drug use and infectious disease. We thus identified the pathway of influence of ACEs in adulthoods and injection of drug use on infectious disease by structural equation models (SEM).

**Methods:**

A cross-sectional study was conducted by respondent driving sampling and consecutive sampling among people who use drugs in southwest of China in 2021. R software 4.2.1 was used to conduct descriptive, univariate, and SEM analysis.

**Results:**

There were 404 participants in total, with an average age of 34 and most males (98.3%) and minorities (79.6%). 95.5% of respondents experienced ACEs with 46.6% of reporting 4 or more ACEs. Correlations in SEM showed that infectious disease might be directly positively affected by injection of drug use (*β* = 0.184), and directly negatively affected by ACEs (*β* = −0.188). Age (*β* = 0.029), Ethnic (*β* = −0.021), Education (*β* = 0.019), Gender (*β* = 0.022), Sex partners (*β* = −0.017), and ACEs (*β* = −0.029) might have indirect effects on infectious disease.

**Conclusion:**

ACEs might be a direct or indirect predictor for infectious disease in adulthood, injection of drug use might be a risk factor and moderate other factors of infection of infectious disease. Strategies for creating a positive home environment, minimizing traumatic or stressful childhood experiences, and increasing awareness of the risks associated with drug injection use are all ways to lower the chances of contracting infectious diseases.

## 1 Introduction

Prevalence of Acquired Immune Deficiency Syndrome (AIDS), Hepatitis B virus (HBV), and Hepatitis C virus (HCV) among drug users were higher especially in the border areas. Approximately 284 million people have used drugs, 14.8 million people who injected drugs (PWID) with 15.2% of HIV infection, 38.8% of HCV and 8.4% of HBV (1). Among PWID, the HIV prevalence was 28 times higher than in the rest of the global adult population with an estimated 68 and 83% condomless sex in the US and Nampula/Nakala, respectively, (2–4). Furthermore, 7.7% of HIV and HCV-positive rates among drug users in China’s border areas thus exceeding the national level of 4.8% (5).

Additionally, poly-drug usage and injection drug use (IDU) were significantly risk factors associated with blood-borne virus diseases that accounted for 43% of future HCV infection up to 79% in high-income countries globally (1, 6–8). Individuals who inject multiple substances exhibited higher levels of risk behaviors and prevalence of sexually transmitted infections (STIs) compared to single users (7, 9), with an increased risk of HIV and Syphilis infection and transmission among men who have sex with men (MSM) (10). Co-infection with syphilis spirochetes was associated with increased HIV viral load and decreased CD4 T-cell counts (11), which HIV infection could impact the normal progression of HBV and HCV (12, 13). Therefore, there existed complex associations between poly-drug uses, IDU and blood-borne viruses’ diseases. More importantly, poly-drug use and IDU may increase the likelihood of engaging in unsafe sexual behaviors like having multiple sexual partners and condomless sex that are major contributors to increased risk of contracting HIV, HCV and syphilis (6, 14, 15). Evidence showed that consistent condom use reduced the risk of contracting the virus by 10–20 times when exposed, compared to inconsistent, non-use or those who had multiple sexual partners (16–18), with an estimated 0.3% risk of HIV transmission and ratios ranging from 2.08 to 2.94 association between stimulant use and unprotected sex and multiple sexual partners (6, 19).

Moreover, adverse Childhood Experiences (ACEs) are associated with risky behaviors related to substance abuse in adulthood with increased risk of HIV and other STIs (20–22). On average, adults were more likely to contract STIs and report association between ACEs and polydrug pattern and alcohol abuse (23, 24), with ACEs being an independent risk of HIV (21, 25). It is essential to explore the pathways through which ACEs affect infectious diseases in adults while controlling for other influencing factors as evidence remains insufficient regarding the associations of ACEs with infectious diseases.

Cross-border activities independently contribute to the increased prevalence of HIV, HBV, HCV, and syphilis. Literature review suggested that cross-border sexual activities were independent predictors of STIs (17), with four times higher HIV prevalence among mobile population than that of the general population in Cambodia, Myanmar, southern China, Vietnam to Thailand with 19.0% HIV-1among Burmese injecting drug users (Yunnan-mIDUs) living in Yunnan (26–28). Migration and mobility were often connected to vulnerability factors like substance use, social exclusion, stigma, and limited access to healthcare or social security services which could increase the chances of HIV transmission (29). However, the relationship among them is not clear. Further exploration of the pathways through which these risk factors act is warranted.

**Figure 1:**
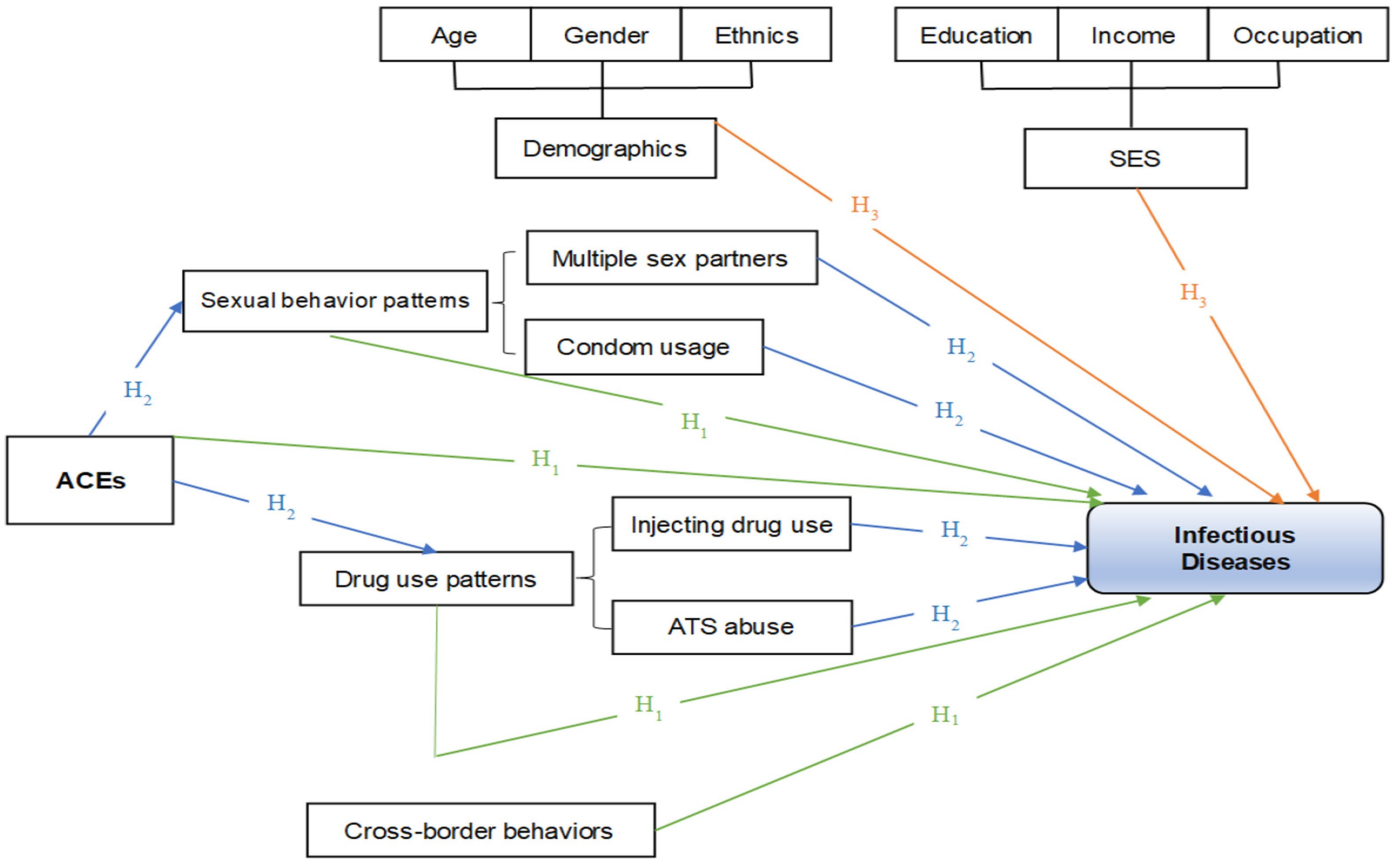
Hypotheses of the study.

While the associations between drug use and infectious diseases are well-established, the potential role of ACEs as both direct and indirect predictors of infection remains insufficiently examined, particularly in under-resourced border regions. We proposed that IDU and ACEs may act as mediating variables in infectious diseases. Structural Equation Modeling (SEM), a more advanced form of regression analysis and strategy (30), was used to simultaneously estimate the direct and indirect pathways from ACEs and IDU to sexually transmitted infectious diseases in adulthood, and to account for latent and observed variable interrelationships. If these intermediary variables can be confirmed, and the causes of the infectious disease pattern are well-documented, future interventions should prioritize addressing early experiences. We hypothesize the following: (1) IDU and ATS use can directly predict infectious diseases in adulthood; (2) having multiple sexual partners and inconsistent condom use can directly predict infectious diseases in adulthood; (3) exposure to ACEs in childhood can directly or indirectly predict infectious diseases in adulthood; (4) cross-border activities can predict infectious diseases (Figure 1).

Our targets aimed to expand upon previous studies and complement them with factors that may contribute to a better understanding of the association between ACEs and drug use. Concretely, we explored the pathways by which ACEs affect infectious diseases in adulthood and the possible moderating effects of injection of drug use in samples from the southwest of China.

## 2 Methods

### 2.1 Study setting

A cross-sectional study was conducted by respondent driving sampling and consecutive sampling among adults who used drugs in Jinghong city, Xishuangbanna Autonomous Prefecture, Yunnan Province from January to July 2021. The inclusion criteria were that participants older than 18 and have used injectable drugs or stimulants during the previous 6 months. Individuals who were unable to participate for medical or psychiatric reasons were omitted. Self-administered questionnaires were primarily used to gather data from eligible participants. For further information on the study setup, participants, sampling, data collection, and processing, refer to our previous publication (31).

### 2.2 Measurements

The questionnaires were used to measure demographics, socio-economic status (SES), cross-border behaviors, ACEs, patterns of sexual behavior, use of illegal addictive drugs and usage patterns. Additionally, the presence of infectious diseases was also collected.

Sex was defined according to external anatomy. Cross-border activity was defined as having experience with going abroad. Sexual behavior patterns included condom use and having multiple sexual partners. Condom usage referred to whether a condom was used during sexual intercourse (vaginal intercourse, oral sex, anal sex, etc.) and multiple sexual partners were identified by the number of sexual partners at the same time, including modes of sexual intercourse with boy/girlfriends, spouses, sex workers, and others. IDU was assessed by asking participants if they had a history of injecting drug use in adulthoods. Infectious disease status was determined based on clinical records indicating physician-diagnosed cases of HIV/AIDS, hepatitis B, hepatitis C, and syphilis. To assess ACEs, a standardized methodology was utilized, incorporating items from the Childhood Trauma Questionnaire (CTQ-SF) (32) and a portion of the items from the USA’s National Survey on Alcohol and Related Conditions-III (33). Nine questions were selected that related to experiences of adverse family events, including neglected daily care, verbal abuse, incidents of sexual abuse, failure to provide medical care, emotional neglect, disapproval, physical violence, an uncaring family climate, and family disruption due to substance use. The questions were adapted to the Chinese context, reviewed and finalized by experts, and pilot tested. Participants were asked to respond to each question on the ACEs questionnaire with either “Yes” or “No.”

### 2.3 Variables

The dependent variable was any HIV/HBV/HCV/Syphilis infection. Demographic variables included age, sex (male = 0, female = 1), ethnicity (Han = 0, minority = 1), and marital status (unmarried = 0, married = 1). SES included occupation (unemployed = 0, employed = 1), education (≤primary school = 0, ≥junior high school = 1), and monthly income. Among them, age and average income were continuous variables. Other factors included cross-border behaviors (non-cross-border = 0, cross-border = 1), condom usage (No = 0, Yes = 1), number of sex partners was continuous variables. Furthermore, ACEs scores varying from 0 to 9 were utilized to assess the overall impact of various ACEs, with higher scores implying more severe exposure to ACEs. It can be fragmented down into four levels: none (zero), mild (1, 2), moderate (3), and severe (equal to or greater than 4).

### 2.4 Data management and statistical analysis

Epi data 3.1 was utilized for data management, and R software 4.2.1 was used for data analysis. For comparison of risk levels in univariate analysis, chi-square and rank sum tests were applied. SEM was employed to investigate direct and indirect effects and associations between variables. A *p*-value below 0.05 was considered statistically significant.

## 3 Results

### 3.1 Distribution of socio-economic-demographics

404 people who used drugs included 397 males and 7 females with mean age of 33.7 years old, 79.5% of ethnic minority, 71.5% of unmarried and employed accounting for 81.4%. 95.5% of respondents experienced ACEs with 46.5% of reporting 4 or more ACEs. 95.5 and 12.4% of respondents were ATS abuser and injection of drug user, respectively. The average values for monthly income and number of sex partner were 3500.0 (2500.0, 5000.0), and 6.0 (3.0, 10.0) respectively. 16.1% of respondents was infected by sexual transmitted diseases (Table 1).

**Table 1:** Socio-economic-demographics and respondents’ variables.

Variables		*N* = 404	%
Age (Mean ± sd.)		33.7 ± 9.8	
Gender	Male	397	98.3
	Female	7	1.7
Ethnicity	Han	83	20.5
	Minority	321	79.5
Marital status	Unmarried	289	71.5
	Married	115	28.5
Education	≤Primary school	206	51.0
	≥Junior school	198	49.0
Occupation	Unemployed	75	18.6
	Employed	329	81.4
Income [Median (P25, P75)]		3500.0 (2500.0, 5000.0)	
Category of ACEs	0	18	4.5
	1–2	105	26.0
	3	93	23.0
	≥4	188	46.5
ATS abuse	No	18	4.5
	Yes	386	95.5
Injection of drug use	No	354	87.6
	Yes	50	12.4
Number of sex partner		6.0 (3.0, 10.0)	
Condom use with temporary sexual partner	No	319	79.0
	Yes	85	21.0
Any HIV/HBV/HCV/Syphilis infection	No	339	83.9
	Yes	65	16.1

### 3.2 Correlations between variables in SEM

Drug injection was positively related to gender (*p* < 0.001), age (*p* < 0.001), education (*p* = 0.05) and infectious diseases (*p* < 0.001) but negatively related to ethnicity (*p* < 0.001), income (*p* < 0.05), ACEs (*p* < 0.001) and ATS abuse (*p* < 0.05). ATS abuse was negatively related to gender (*p* < 0.01), age (*p* < 0.01), marriage (*p* < 0.05) and infectious disease (*p* < 0.01) but positively related to ACEs (*p* < 0.001). Infectious disease was positively related to gender (*p* < 0.01) and age (*p* < 0.05) but negatively related to ACEs (*p* < 0.001) (details refer to Table 2).

**Table 2:** Correlations between variables in SEM model.

	Gender	Age	Ethnicity	Marriage	Education	Occupation	Income	ACEs	ATS abuse	Drug injection	Condom use	Sex partner	Cross border	Infectious diseases
Gender	1	0.136**	−0.073	0.042	0.06	−0.132**	−0.099*	−0.155**	−0.155**	0.181***	−0.069	−0.052	−0.046	0.148**
	—	0.001	0.141	0.395	0.232	0.008	0.020	0.002	0.002	0.000	0.169	0.221	0.356	0.003
Age	0.136**	1	−0.054	0.245**	−0.203***	0.025	−0.149***	−0.123**	−0.123**	0.161***	−0.005	−0.053	0.028	0.090*
	0.001	—	0.188	0.000	0.000	0.551	0.000	0.003	0.003	0.000	0.900	0.130	0.502	0.029
Ethnicity	−0.073	−0.054	1	−0.046	−0.200***	0.230***	−0.067	0.187***	−0.05	−0.181***	0.007	−0.009	−0.039	0.006
	0.141	0.188	—	0.358	0.000	0.000	0.116	0.000	0.311	0.000	0.889	0.841	0.432	0.906
Marriage	0.042	0.245***	−0.046	1	−0.081	0.09	−0.074	−0.05	−0.103*	0.079	−0.097	−0.064	0.027	0.037
	0.395	0.000	0.358	—	0.105	0.072	0.084	0.317	0.039	0.111	0.052	0.131	0.586	0.454
Education	0.06	−0.203***	−0.200***	−0.081	1	−0.232***	0.096*	−0.004	0.068	0.098*	−0.069	0.06	0.067	0.042
	0.232	0.000	0.000	0.105	—	0.000	0.024	0.932	0.174	0.050	0.168	0.159	0.179	0.395
Occupation	−0.132**	0.025	0.230***	0.09	−0.232**	1	0.051	0.02	−0.011	−0.091	−0.05	−0.01	−0.088	−0.033
	0.008	0.551	0.000	0.072	0.000	—	0.230	0.683	0.832	0.067	0.313	0.812	0.077	0.501
Income	−0.099*	−0.149***	−0.067	−0.074	0.096*	0.051	1	0.053	0.074	−0.085*	0.052	0.076*	0.075	−0.054
	0.020	0.000	0.116	0.084	0.024	0.230	—	0.214	0.084	0.047	0.224	0.037	0.077	0.203
ACEs	−0.155**	−0.123**	0.187***	−0.05	−0.004	0.02	0.053	1	0.244***	−0.210***	0.053	0.054	−0.041	−0.232***
	0.002	0.003	0.000	0.317	0.932	0.683	0.214	—	0.000	0.000	0.291	0.204	0.412	0.000
ATS abuse	−0.155**	−0.123**	−0.05	−0.103*	0.068	−0.011	0.074	0.244***	1	−0.101*	0.053	0.02	0.064	−0.134**
	0.002	0.003	0.311	0.039	0.174	0.832	0.084	0.000	—	0.043	0.291	0.629	0.201	0.007
Drug injection	0.181**	0.161***	−0.181***	0.079	0.098*	−0.091	−0.085*	−0.210***	−0.101*	1	0.027	−0.02	0.047	0.224***
	0.000	0.000	0.000	0.111	0.050	0.067	0.047	0.000	0.043	—	0.584	0.642	0.340	0.000
Condom use	−0.069	−0.005	0.007	−0.097	−0.069	−0.05	0.052	0.053	0.053	0.027	1	0.204***	0.084	−0.094
	0.169	0.900	0.889	0.052	0.168	0.313	0.224	0.291	0.291	0.584	—	0.000	0.093	0.060
Sex partners	−0.052	−0.053	−0.009	−0.064	0.06	−0.01	0.076*	0.054	0.02	−0.020	0.204***	1	0.046	0.024
	0.221	0.130	0.841	0.131	0.159	0.812	0.037	0.204	0.629	0.642	0.000	—	0.281	0.572
Cross border	−0.046	0.028	−0.039	0.027	0.067	−0.088	0.075	−0.041	0.064	0.047	0.084	0.046	1	0.005
	0.356	0.502	0.432	0.586	0.179	0.077	0.077	0.412	0.201	0.340	0.093	0.281	—	0.913
Infectious diseases	0.148**	0.090*	0.006	0.037	0.042	−0.033	−0.054	−0.232***	−0.134**	0.224***	−0.094	0.024	0.005	1
	0.003	0.029	0.906	0.454	0.395	0.501	0.203	0.000	0.007	0.000	0.060	0.572	0.913	—

*p < 0.05, **p < 0.01, ***p < 0.001.

### 3.3 Predictors of infectious disease by SEM

**Figure 2:**
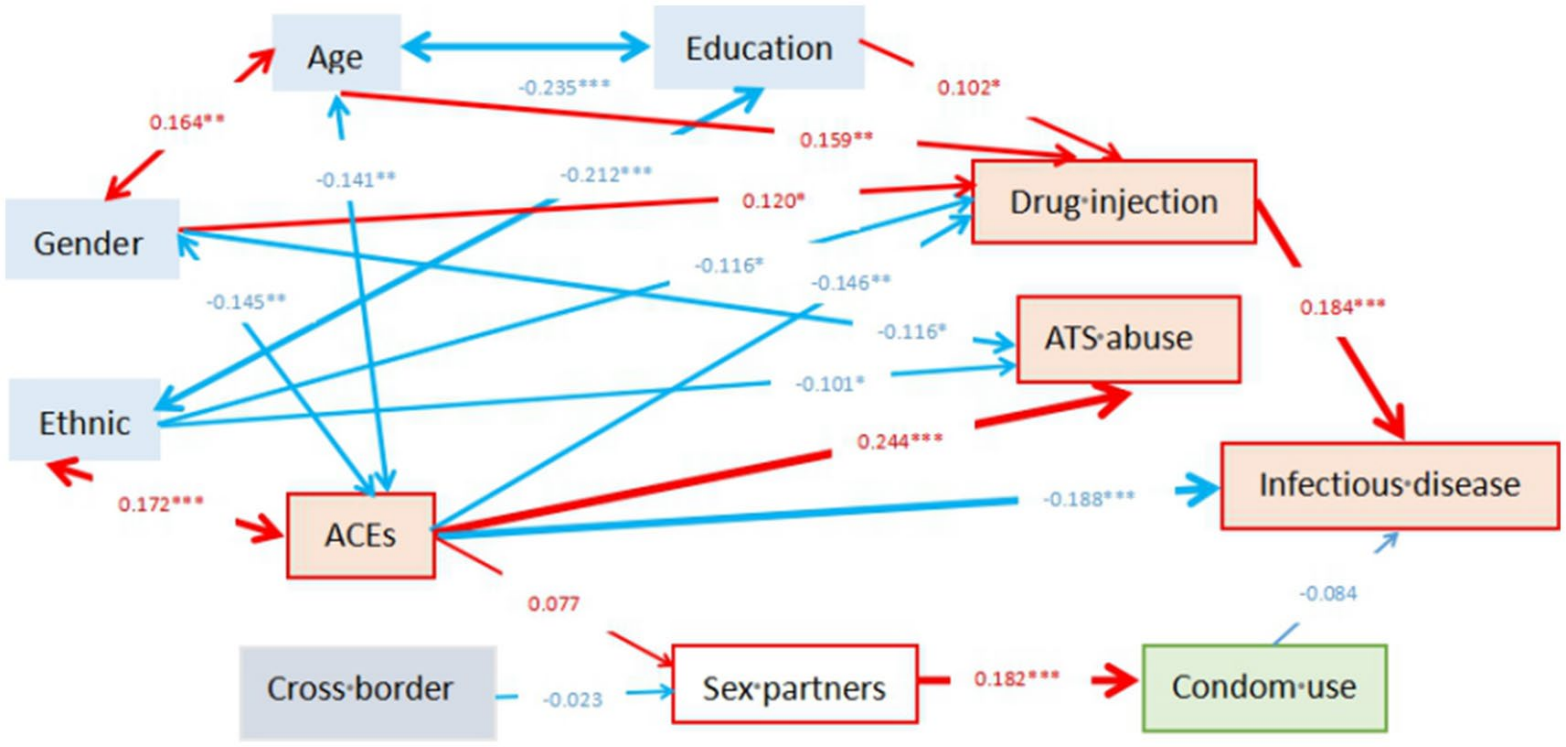
Pathways illustrating the effects of adverse childhood experiences and drug injection on infectious diseases among drug-using adults. Parameters in this figure are standardized value. The significant level for path coefficients was set at *p < 0.05, **p < 0.01, ***p < 0.001.

The final structural model fitted the current data well, Chi-square = 41.828 (df = 34, *p* = 0.167), Root Mean Square Error of Approximation (RMSEA) = 0.024 (90% CI: 0.000, 0.045), Adjusted Goodness of Fit Index (AGFI) = 0.962, and Comparative Fit Index (CFI) = 0.962. Figure 2 showed that Infectious disease was positively affected by drug injection (*β* = 0.184), and negatively affected by ACEs (*β* = −0.188). Drug injection was positively associated with education (*β* = 0.102), age (*β* = 0.159), gender (*β* = 0.120) and negatively associated with ACEs (*β* = −0.146) and ethnic (*β* = −0.116). ATS abuse was positively affected by ACEs (*β* = 0.244) and negatively associated with gender (*β* = −0.166) and ethnic (*β* = −0.101). The details of other associations between variables studied were showed in Figure 2.

## 4 Discussion

Exposure to ACEs in childhood could predict infectious diseases in adulthood through both direct and indirect negative effects on infectious diseases by positively impacting the adult injection of drug use. Meanwhile, drug use injection also had a direct positive effect on infectious diseases while condom use was not associated with infectious diseases. Delving into demographics, it turns out that age, gender, ethnicity and education might subtly influence infectious disease by shifting behaviors related to injecting drug use.

Our findings support the first hypothesis that IDU was directly associated with infectious diseases and a higher risk of HIV and HCV prevalence which was consistent with other studies (1, 34–36). Nevertheless, studies have also showed 30–40% were not exposed to HCV in high-risk settings with IDU (37). There was a high risk of contracting a blood-borne virus through IDU as the use of contaminated needles, syringes, or drug injection equipment was the main cause of HIV and HCV infection (34). The Xishuangbanna region is traditionally an area of high rate of IDU, the transmission of infection through unprotected sexual intercourse may sometimes be ignored in the context of infectious diseases caused by IDU.

ACEs exposure could predict infectious diseases in adulthood, through both indirect and direct negative effects on infectious diseases by positively affecting adult injection of drug use. Previous findings that ACEs were associated with poor health outcomes in adulthood, such as increased risk of HIV and other sexually transmitted diseases (22, 38, 39). Whereas our study found ACEs contributed to a directly negative effect on sexually transmitted infections. One possible explanation for this finding is the concept of “benign resilience,” an unquantified resilience mechanism that mitigates the long-term effects of ACEs and reduces the likelihood of risky behaviors in adulthood, thereby reducing the risk of infectious diseases (38, 40). And, in Anhui Province, China, 44.6% of methamphetamine users reported negative childhood events (41). On the other hand, ACEs indirectly predict infectious diseases through drug injection as a mediator in our study. Firstly, drug injection was negatively associated with ACEs. In contrast, other scholars found that IDU in adulthood was strongly associated with ACEs, and that early childhood maltreatment led to increased risk of substance abuse, as well as being a possible victim and perpetrator of HIV risk behaviors (42–44). The characteristic of individuals with ACEs to holistically avoid negative health and social outcomes associated with childhood was called resilience, which was said to reduce the adverse effects (45). The target population of this study may establish the negative outcomes associated with drug injection while growing up, thereby reducing the likelihood of IDU in adulthood. Moreover, ACEs exposure did not indirectly predict infectious diseases by condom use as a mediator, which was totally contrary with our hypothesis. Previous studies have shown that ACEs were related to a lack of condom use and a higher number of sexual partners (46, 47). The possible reason may be the low detection rate of infectious diseases in the target population of this study. Notably, these infectious diseases can alter the immune system, leading to more severe health consequences and disease burdens in the long effects. It needs more explore the long effects rather than the relationship between ACEs and infectious diseases. One other possible explanation is that the presence of a suppression effect. Suppression occurs when the inclusion of a mediating or confounding variable in the model changes the direction or magnitude of the direct relationship between the independent and dependent variables.

Education, age, and gender might be positively associated with injecting drug use. Age and education exhibited correlation, as older adults tend to have lower levels of education, possibly due to economic or developmental reasons that prevented them from receiving education in their youth. Furthermore, older and better educated individuals were more likely to engage in drug injection, which aligns with other studies that have shown higher rates of blood-borne virus infections among older adults with a history of injecting drug use (8). Moreover, women may have a higher likelihood of injecting drugs and subsequently developing infectious diseases, while men with ACEs were also more prone to drug injection. Previous research has indicated that women were more susceptible to be affected by multiple forms of abuse and poor mental health outcomes that later manifest as IDU (42). These findings suggest that women may be at elevated risk for both ACE exposure and subsequent high-risk behaviors such as IDU, which can in turn increase vulnerability to infectious diseases. While the sample was male-dominated overall, the pathways observed in the model underscore the importance of addressing gender-specific trauma and substance use trajectories in prevention efforts. Ethnicity showed a negative association with drug injection, which may be attributed to the fact that the study area was predominantly inhabited by ethnic minority populations. The relatively lower rates of injection among these groups could reflect cultural or social protective factors, whereas Han Chinese individuals, though fewer in number locally, may experience increased psychosocial stressors leading to higher vulnerability to IDU.

On the other hand, the study did not provide enough evidence to validate our second hypothesis regarding the relationship between sexual behavior and infectious diseases. Although sexual risk factors were not associated with the HIV epidemic, majority (73%) of adult stimulant users in the United States involved in risky sexual behaviors with only 38% consistent condoms use (48). Conversely, another study alluded that an increase in the number of condomless anal sex partners could explain why MSM and those who primarily injected methamphetamine were more likely to be diagnosed with syphilis and HIV, and association between women who engage in sex works and condom use more often (7). Meanwhile, the association between cross-border behavior and infectious diseases also could not be elucidated. This may be because cross-border behavior, although common in border areas and with frequent cross-border marriages, was not a significant mobility factor due to the presence of stable, long-term residence in one village per country. A previous study also showed no significant differences in HIV, HCV, and syphilis infection rates between Chinese and Myanmar nationalities, possibly indicating that the high-risk sexual behaviors among individuals in cross-border marriages do not differ significantly in the long term. This observation may also be influenced by local infectious disease prevention and control policies, such as the equal entitlement of foreign women in cross-border marriages to resident health insurance and basic public health services (49).

There were several weaknesses in this study. Firstly, ACEs were assessed via self-recall, which could introduce information bias. Secondly, the cross-sectional design could hamper the ability to derive causation. Furthermore, SEM revealed pathways that were not comprehensive, as there were few directly observed variables and no potential variables considered that could influence exposure and outcome, thus limiting causal interpretation. Lastly, the study was confined to drug users receiving treatment, lacking a control group of non-drug users, and the majority of participants were Dai male patients, thereby restricting the generalizability of the findings.

## 5 Conclusion

ACEs might be a direct or indirect predictor for infectious disease in adulthood, injection of drug use might be a risk factor and moderate other factors of infectious disease. Subsequent cohort studies should be conducted to further explore the causal relationship between them.

## Data Availability

The raw data supporting the conclusions of this article will be made available by the authors, without undue reservation.
